# Advances in the Treatment of Yaws

**DOI:** 10.3390/tropicalmed3030092

**Published:** 2018-08-29

**Authors:** Michael Marks

**Affiliations:** Clinical Research Department, London School of Hygiene & Tropical Medicine, Keppel Street, London WC1E 7HT, UK; michael.marks@lshtm.ac.uk; Tel.: +44-20-7636-8635

**Keywords:** yaws, *Treponema pallidum*

## Abstract

Yaws is one of the three endemic treponematoses and is recognised by the World Health Organization as a neglected tropical disease. Yaws is currently reported in 15 countries in the Pacific, South-East Asia, West and Central Africa, predominantly affects children, and results in destructive lesions of the skin and soft tissues. For most of the twentieth century penicillin-based treatment was the standard of care and resistance to penicillin has still not been described. Recently, oral azithromycin has been shown to be an effective treatment for yaws, facilitating renewed yaws eradication efforts. Resistance to azithromycin is an emerging threat and close surveillance will be required as yaws eradication efforts are scaled up globally.

## 1. Introduction

Yaws, caused by *Treponema pallidum* subsp. *pertenue*, is one of the three endemic treponematoses (along with *T.p.* subsp. *endemicum*, the causative agent of bejel, and *T. carateum*, the causative agent of pinta) and is recognised by the World Health Organization as a neglected tropical disease (NTD) [1,2]. The disease predominantly affects children and results in destructive lesions of the skin and soft tissues. Yaws is still found in Africa, South-East Asia and the Pacific. *T.p.* subsp. *pertenue* is closely related to *T.p.* subsp. *pallidum*, the causative agent of syphilis [3] but neither sexual nor mother-to-child transmission of yaws is believed to occur [2].

## 2. Epidemiology

Yaws is currently reported in 15 countries in the Pacific, South-East Asia, West and Central Africa [4,5,6,7,8,9]. Currently the most cases are reported in Papua New Guinea, the Solomon Islands and Ghana and all have reported in excess of 15,000 cases in recent years. In the mid-twentieth century, yaws was endemic in South America and the Caribbean, but control programmes in the mid-twentieth century (see below) are believed to have eliminated yaws from the majority of countries in the region [10,11]. India interrupted transmission in 2004 and declared elimination in 2006 [12] following a sustained programme that began in 1996. 

Yaws is restricted to warm and humid environments [13]. Transmission is skin to skin contact from active infectious lesions [14]. Early studies had suggested that flies may play a role in transmission but there is no definitive proof that this occurs [14,15,16]. The majority of active infections cases occur in children aged under 15 years. Closely-related treponemal infections have been identified in primate populations, but zoonotic transmission to humans has not been established [17,18].

## 3. Clinical Features

As with other treponemal infections, yaws is characterized by a multi-stage disease process predominantly involving skin, bones and cartilage.

The initial stage of primary yaws is the development of an erythematous lesion that occurs at the site of inoculation after an incubation period of 9–90 days [14,19]. These lesions may break down forming an ulcerated plaque over a period of 1–2 weeks. Primary lesions occur most frequently on the lower limbs or buttocks [13,20], whilst genital lesions are extremely uncommon. If patients are left untreated then primary lesions will heal spontaneously, with scarring, over a period of 3–6 months [21].

Without treatment individuals will progress to secondary yaws. Secondary yaws predominantly involves the skin and bones [20,22]. As with secondary syphilis, both generalised lymphadenopathy and constitutional symptoms, including fever and malaise, are frequently reported in patients with secondary yaws [19]. A wide-range of skin manifestations occur in secondary yaws including macular lesions and hyperkeratotic lesions on the palms and soles (‘crab yaws’) [14,19,20]. Osteoperiostitis, affecting the fingers (resulting in dactylitis) or long bones (forearm, fibula, tibula) is the most common bony manifestation of secondary yaws. Following treatment of early yaws (primary or secondary) skin lesions usually resolve within 2–4 weeks and bone pain may start to resolve in as little as 48 h [23].

In the absence of treatment of early yaws, patients go on to develop latent infection. During this clinical stage of disease patients have reactive serology but no clinical evidence of infection. Relapse from latent to active disease can occur for a period of up to 5–10 years and serve as a source of onward transmission. Previously up to 10% of patients were reported to develop the late-stage manifestations of tertiary yaws, but this is now much less commonly reported [19]. Tertiary disease may manifest as gummatous nodules resulting in tissue necrosis, a destructive osteitis which can cause destruction of the maxilla (gangosa), or bowing of the shins (sabre shin), or a hypertrophic periostitis causing exostosis of the paranasal maxilla (gondou). 

Clinical diagnosis is supported by serological testing. Diagnostic testing combines a treponemal assay (such as the *Treponema pallidum* particle agglutination assay) and a non-treponemal assay (such as the rapid plasma regain assay). Treponemal tests are more specific but remain positive for life. The titre of non-treponemal assays rises following infection and falls following treatment. A four-fold fall in titre (e.g., from 1:16 to 1:4) is considered consistent with serological cure. Newer point-of-care treponemal and non-treponemal assays have increasingly taken on the role of traditional lab-based assays [24,25].

An important recent discovery has been the finding that *Haemophilus ducreyi* may cause ulcerative skin lesions similar to those seen in patients with early yaws [26,27]. *H. ducreyi* may cause lesions in both patients who have non-reactive and reactive serological tests for yaws, which complicates assessment of patients with suspected active yaws as serological point of care tests cannot be used to accurately provide a definitive diagnosis. When molecular tests are used, a large proportion of patients suspected to have active early yaws are found in fact to have latent yaws (reactive serological tests) and a different cause for the current skin lesion. 

## 4. Penicillin-Based Treatment

Early studies conducted in Haiti demonstrated that penicillin-based treatments were highly efficacious in the treatment of yaws [23] and penicillin-based therapy was subsequently adopted as the standard of care worldwide for all endemic treponematoses [14]. Different penicillin-based regimes are effective for the treatment of yaws, but long-acting intramuscular benzathine benzylpenicillin was the most commonly used regimen. Lower doses were used than those recommended for the treatment of venereal syphilis (1.2 million units for the treatment of adults and 0.6 million units for the treatment of children) [14]. Although there have been rare reports of ‘treatment failure’ with penicillin-based therapy, the inability to distinguish treatment failure from reinfection makes these data extremely difficult to interpret, especially in highly endemic settings [28]. As with syphilis, no evidence of penicillin resistance has developed in yaws despite it being the first-line treatment for more than 50 years.

During the mid-twentieth century, successful treatment campaigns were conducted targeting yaws and the other endemic treponematoses [29]. In 1949, the World Health Assembly passed a resolution supporting efforts for the control and elimination of the endemic treponematoses, including yaws. WHO and UNICEF led a global effort between 1952 and 1964, based on penicillin treatment. At the time, the recommended strategy varied, based on the prevalence of active yaws in the community (Table 1). Although not ultimately successful in achieving eradication, the programme did significantly reduce the global prevalence of yaws by as much as 98% [30]. Following these efforts, the incidence of the disease rebounded in a number of countries in the 1970s, ultimately leading to a further World Health Assembly resolution in 1978, which resulted in some countries renewing control efforts [29].

## 5. Azithromycin-Based Treatment

Despite its efficacy, treatment with penicillin remains challenging in many settings. Injectable penicillin requires access to a secure cold chain and trained staff to administer therapy, both of which are not always available in the remote locations where yaws is found. Treatment also carried a small but important risk of anaphylaxis. Supplies of benzathine benzylpenicillin have also been insecure in recent years [31], with implications for reliable access to treatment. 

Azithromycin, an oral macrolide antibiotic, had previously been demonstrated to be effective in the treatment of syphilis [32] with cure rates equivalent to those of benzathine benzylpenicillin. Given the high degree of genetic homology between syphilis and yaws, azithromycin was an attractive option for study as an alternative treatment for yaws. In a landmark study conducted in Papua New Guinea, patients with early (primary and secondary) yaws were randomised to receive treatment with either a single dose of intramuscular benzathine benzylpenicillin or a single, oral dose of azithromycin. Azithromycin was shown to be non-inferior to benzathine benzylpenicillin with a cure rate greater than 95% in both arms [33]. Subsequently, a study conducted in Ghana also confirmed the non-inferiority of azithromycin compared to benzathine benzylpenicillin [34].

These initial studies all enrolled patients with early active yaws and used a dose of 30 mg/kg (max. 2 g). Two outstanding questions were whether azithromycin was effective for the treatment of latent infection and whether the lower dose of azithromycin used for the treatment of trachoma (20 mg/kg—max. 1 g) was also efficacious in the treatment of yaws. In a longitudinal cohort study in Papua New Guinea, treatment with azithromycin was demonstrated to have a cure rate for patients with latent yaws equivalent to the cure rate of patients with active yaws [35]. The efficacy of the low versus standard dose azithromycin was compared in a randomised control trial in patients with both early active and latent yaws, conducted in Papua New Guinea and Ghana. The clinical cure rate was equivalent in both low and standard dose arms. The serological cure rate in patients with active yaws was slightly lower in the low-dose arm. In patients with latent yaws the serological cure rate was equivalent with both doses of azithromycin [36]. Taken together with observational data, this suggests that low-dose azithromycin is also effective in the treatment of yaws, although the 30 mg/kg dose remains the standard of care.

Unlike with penicillin, resistance to azithromycin is well described in *T. pallidum*. Resistance is mediated by one of two mutations in the 23s rDNA and can be detected by specific molecular tests [37,38,39,40,41]. In the context of large scale azithromycin-based treatment programmes (see below), treatment failure and genotypic resistance to azithromycin has now also been described in yaws [42].

## 6. Community Treatment with Azithromycin

Azithromycin has been widely distributed at the community level for the treatment of trachoma [43] and the WHO recommends mass drug administration (MDA) of azithromycin for the elimination of trachoma as a public health problem as part of the SAFE (Surgery, Antibiotics, Facial cleanliness and Environmental improvement) strategy [44]. MDA of azithromycin has been shown to be safe and indeed there is considerable evidence that MDA may result in significant off-target benefits including reductions in child mortality [45].

Taken together, the evidence of the safety of MDA with azithromycin and the efficacy of azithromycin for the treatment of yaws provide the rationale behind the revised WHO strategy for the eradication of yaws (the Morges strategy). This strategy emphasises MDA of azithromycin in communities where yaws is endemic. Following an initial round of MDA (referred to as total community treatment—TCT) it may be appropriate to conduct further MDA or to switch to a strategy of treating active cases and their contacts (total targeted treatment—TTT) [46]. No clear evidence currently exists to guide the decision regarding when to switch between TCT and TTT, but modelling studies suggest that TCT is a preferable strategy because it ensures a higher coverage of latent yaws cases [47,48].

Initial assessments of the efficacy of azithromycin MDA were conducted in both the Pacific and West Africa. In a study in the Solomon Islands, communities received a single round of MDA of azithromycin (conducted for the elimination of trachoma as a public health problem) at a dose of 20 mg/kg (max. 1g) and were followed up at 6 and 18 months following MDA [49,50,51]. A significant reduction in the prevalence of both active and latent yaws was seen at both 6 and 18 months following MDA. In Ghana, MDA with azithromycin (30 mg/kg, max. 2 g) was conducted in a single district and follow-up conducted at 12 months. As in the Solomon Islands, a significant reduction in both active and latent yaws was documented [52]. 

The most comprehensive evaluation of the WHO Morges strategy was conducted in Lihir, Papua New Guinea [53]. In this study of more than 15,000 individuals, an initial round of mass treatment was undertaken, followed by six-monthly rounds of surveillance and treatment of new cases and their contacts. In keeping with the studies discussed above, this study demonstrated a marked reduction in the prevalence of both active and latent yaws. Despite this initial success, interruption of transmission was not achieved [42]. Ongoing transmission was driven both by cases imported from outside the study site and cases arising in individuals missed during the initial mass treatment phase of the study. Thirty-six months into the study, a single case of treatment failure was detected. The patient had been treated at 30 months with azithromycin but at month 36 had clinical evidence of progression and serological evidence of treatment failure. Subsequent molecular testing confirmed genotypic azithromycin resistance in this case. Alongside the index case, several contacts were detected at month 36 and month 42 of the study with azithromycin-resistant yaws. Although subsequent treatment with benzathine benzylpenicillin was used to successfully ring-fence the outbreak, these data highlight the risk of emerging azithromycin resistance threatening yaws eradication efforts [42].

## 7. Conclusions

There has been considerable progress in the treatment of yaws in the last decade. Azithromycin has emerged as an effective and easily deliverable oral treatment option that can be used to treat both individual cases and during community MDA. The emergence of azithromycin resistance highlights the need for ongoing surveillance to support yaws eradication efforts globally. Further studies are needed to better define the optimum MDA strategy including the number of rounds and population coverage required to interrupt the transmission of yaws.

## Figures and Tables

**Table 1:** Historical strategy for the eradication of yaws: 1952–1964.

Prevalence of Clinically Active Yaws	Treatment Strategy
Hyperendemic: above 10%	Benzathine benzylpenicillin to the whole community (total mass treatment)
Mesoendemic: 5–10%	Treat all active cases, all children under 15 and all contacts of infectious cases (juvenile mass treatment)
Hypoendemic: under 5%	Treat all active cases and all household and other contacts (selective mass treatment)
